# COX-2/mPGES-1/PGE_2_ cascade activation mediates uric acid-induced mesangial cell proliferation

**DOI:** 10.18632/oncotarget.14363

**Published:** 2016-12-29

**Authors:** Shuzhen Li, Zhenzhen Sun, Yue Zhang, Yuan Ruan, Qiuxia Chen, Wei Gong, Jing Yu, Weiwei Xia, John Ci-Jiang He, Songming Huang, Aihua Zhang, Guixia Ding, Zhanjun Jia

**Affiliations:** ^1^ Department of Nephrology, Children's Hospital of Nanjing Medical University, Nanjing 210008, China; ^2^ Jiangsu Key Laboratory of Pediatrics, Nanjing Medical University, Nanjing 210029, China; ^3^ Nanjing Key Laboratory of Pediatrics, Children's Hospital of Nanjing Medical University, Nanjing 210008, China; ^4^ Department of Endocrinology, Jiangsu Province Hospital of TCM, Affiliated Hospital of Nanjing University of TCM, Nanjing 210008, China; ^5^ Division of Nephrology, Department of Medicine, Mount Sinai School of Medicine, New York, NY 210029, USA

**Keywords:** uric acid, COX-2, mPGES-1, PGE_2_, mesangial cells

## Abstract

Hyperuricemia is not only the main feature of gout but also a cause of gout-related organ injuries including glomerular hypertrophy and sclerosis. Uric acid (UA) has been proven to directly cause mesangial cell (MC) proliferation with elusive mechanisms. The present study was undertaken to examined the role of inflammatory cascade of COX-2/mPGES-1/PGE_2_ in UA-induced MC proliferation. In the dose- and time-dependent experiments, UA increased cell proliferation shown by the increased total cell number, DNA synthesis rate, and the number of cells in S and G2 phases in parallel with the upregulation of cyclin A2 and cyclin D1. Interestingly, UA-induced cell proliferation was accompanied with the upregulation of COX-2 and mPGES-1 at both mRNA and protein levels. Strikingly, inhibition of COX-2 via a specific COX-2 inhibitor NS-398 markedly blocked UA-induced MC proliferation. Meanwhile, UA-induced PGE_2_ production was almost entirely abolished. Furthermore, inhibiting mPGES-1 by a siRNA approach in MCs also ameliorated UA-induced MC proliferation in line with a significant blockade of PGE_2_ secretion. More importantly, in gout patients, we observed a significant elevation of urinary PGE_2_ excretion compared with healthy controls, indicating a translational potential of this study to the clinic. In conclusion, our findings indicated that COX-2/mPGES-1/PGE_2_ cascade activation mediated UA-induced MC proliferation. This study offered new insights into the understanding and the intervention of UA-related glomerular injury.

## INTRODUCTION

Uric acid (UA) is an intermediary product of the purine degradation pathway in cells [1]. Urate is freely filtered, reabsorbed, and secreted at the kidneys [2]. Kidneys are responsible for the excretion of two-thirds of the daily uric acid, with the remaining one-third being excreted through the gastrointestinal tract. Hyperuricemia is defined as a serum uric acid level >416.5 μM in males and > 357 μM in females [3]. Emerging evidence highly suggested that hyperuricemia was associated with the occurrence and progression of kidney injury in patients with gout [4, 5]. Patients with hyperuricemia often have glomerular hypertrophy and tubule interstitial injury independent of intrarenal crystal formation [6, 7]. Studies have shown that hyperuricemia could accelerate renal disease in the remnant kidney model and cyclosporine nephropathy [4, 7, 8]. *In vitro*, UA was found to directly induce glomerular mesangial cell (MC) proliferation [9]. Thus, the UA-related mesangial cell proliferation might contribute to the glomerular hypertrophy and sclerosis, which could finally result in the development and progression of renal injury. However, the detailed mechanism of UA-associated mesangial cell proliferation is still elusive, leading to the lack of effective targets in the prevention and treatment of hyperuricemia-related kidney disease.

PGE_2_ is a known inflammatory mediator and contributed to pathogenesis of many diseases including renal injury, such as vascular smooth muscle tonus, glomerular filtration, renin release and tubular salt and water transport [10, 11]. In general, inducible enzymes of COX-2 and mPGES-1 are responsible for the PGE_2_ generation under disease conditions, while COX-1 and another two PGE_2_ synthases (mPGES-2 and cPGES) contribute to the basal levels of PGE_2_ in various tissues [12]. A recent study indicated that COX-2 was related to UA-activated proliferation of smooth muscle cells possibly via TXA2 [7]. Another study reported that PGE_2_ played an important role in mediating transforming growth factor (TGF)-β_1_-induced mesangial cell damage [13]. However, the role of COX-2/mPGES-1/PGE_2_ cascade activation in UA-induced mesangial cell proliferation was not defined. In the present study, employing the siRNA and pharmacological strategies, we fully investigated the activation and contribution of this inflammatory cascade in mesangial cell proliferation induced by UA.

## RESULTS

### UA induced MC proliferation

**Figure 1:**
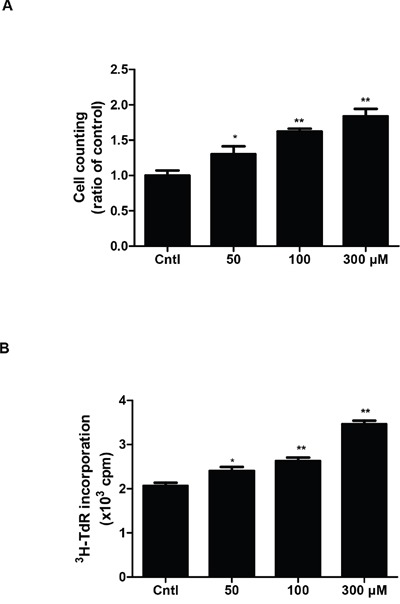
Effect of uric acid on cell proliferation in mouse mesangial cells **A** & **B.** After mesangial cells were cultivated to 60%–70% confluence, they were treated with uric acid at different doses (0, 50, 100, 300 μM) for 24 h and the cell proliferation was determined by cell counting (A) and [3H] thymidine (3H-TdR) incorporation (B). Values are means ± SE; n = 6 for each group. * *P*< 0.05 vs. control, ** *P*< 0.01 vs. control.

To investigate whether UA could induce MC proliferation in the present study, we treated MCs with UA at different doses. Cell proliferation was examined by direct cell counting and DNA synthesis rate ([^3^H] thymidine uptake). UA treatment at 50, 100 and 300 μM for 24 h gradually increased cell numbers in a dose-dependent manner (Figure 1A). To further confirm this result, we examined DNA synthesis rate. Similarly, the amount of [^3^H] thymidine uptake in UA-treated MCs was also elevated in a dose-dependent manner (Figure 1B).

### UA induced cell cycle progression in MCs

**Figure 2:**
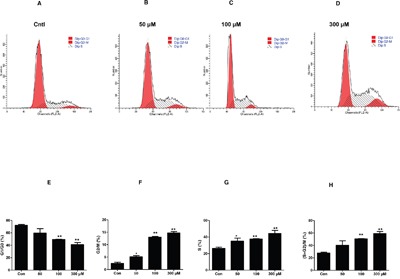
Effect of uric acid on cell cycle progression in mouse mesangial cells The percentage of cells at different cell cycle phases was detected by flow cytometry after mesangial cells were treated with the indicated doses of uric acid for 24 h. **A-D.** Representative images of cell cycle with different doses of uric acid. **E-H.** Percentage of cells at S, G1/G0, and (S + G2)/M phases. Values are means ± SE; n = 6 for each group. * *P*< 0.05 vs. control, ** *P*< 0.01 vs. control.

In order to confirm the MC proliferation shown above, we analyzed cell cycle by flow cytometry in MCs exposed to different dose of UA. As shown by data, UA caused a significant decrease of MC numbers in the G1/G0 phase but an increase of cell numbers in the S phase (Figure 2A-2H), indicating that UA can trigger the cell cycle progression in MCs.

### UA enhanced the expressions of cyclin D1 and cyclin A2 in MCs

**Figure 3:**
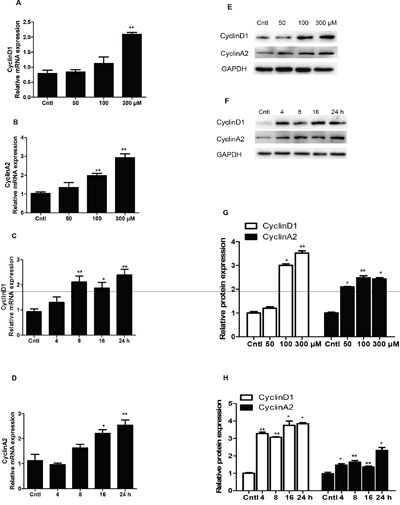
Effects of uric acid on the expressions of cyclin D1 and cyclin A2 in mesangial cells **A** & **B.** After mesangial cells were treated with uric acid (50-300 μM) for 24 h, a dose-dependent increase of cyclin D1 (A) and cyclin A2 (B) mRNA levels was observed. **C** & **D.** Uric acid at 300 μM caused a time-dependent induction of cyclin D1 and cyclin A2 mRNA expressions. Protein levels of cyclin D1 and cyclin A2 were also elevated in dose- and time-dependent manners. **E.** Representative images of cyclin D1 and cyclin A2 Western blots in dose-dependent experiments. **F.** Representative images of cyclin D1 and cyclin A2 Western blots in time-dependent experiments. **G** & **H.** Quantification of Western blots in E (G) and F (H). All values are means ± SE; n = 6 for each group. * *P*< 0.05 vs. control, ** *P*< 0.01 vs. control.

qRT-PCR and Western blotting analysis of cyclins showed that UA strikingly increased the mRNA and protein levels of cyclin D1 and cyclin A2 in dose- and time-dependent manners (Figure 3A-3H). These results indicated that cyclin D1 and cyclin A2 were significantly elevated by UA, which could be of importance in modulating the cell cycle progression in the current experimental setting.

### UA treatment significantly upregulated COX-2 and mPGES-1 expression in MCs

**Figure 4:**
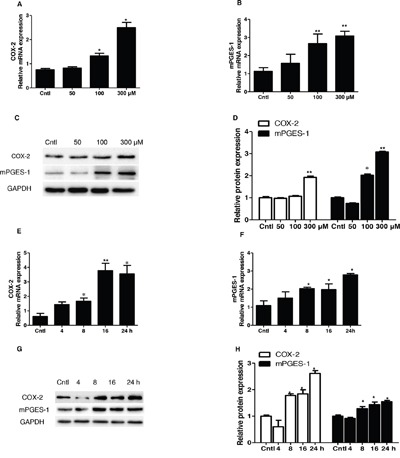
Expressions of COX-2 and mPGES-1 in uric acid-treated mesangial cells Mesangial cells were treated with the indicated doses of uric acid (0, 50, 100, 300 μM) for 24 h and then COX-2 and mPGES-1 mRNA and protein expressions **A-D.** were analyzed by qRT-PCR and Western blotting. A time course analysis of COX-2 and mPGES-1 expression following uric acid treatment at a dose of 300 μM was also examined **E-H.** Values are means ± SE; n = 6 for each group. * *P*< 0.05 vs. control, ** *P*< 0.01 vs. control.

To study the potential pathogenic mechanism involved in UA-induced MC proliferation, COX-2 and mPGES-1 expressions were detected by Western blotting and qRT-PCR. Strikingly, COX-2 and mPGES-1 were dose-dependently elevated at both mRNA and protein levels (Figure 4A-4D). With a treatment of 300 μM UA, we also found a time-dependent induction of COX-2 (Figure 4E) and mPGES-1 (Figure 4F) at both mRNA and protein levels (Figure 4G-4H). These results indicated that COX-2 and mPGES-1 could be directly induced by UA in MCs.

### Inhibiting COX-2 blocked UA-induced MC proliferation

**Figure 5:**
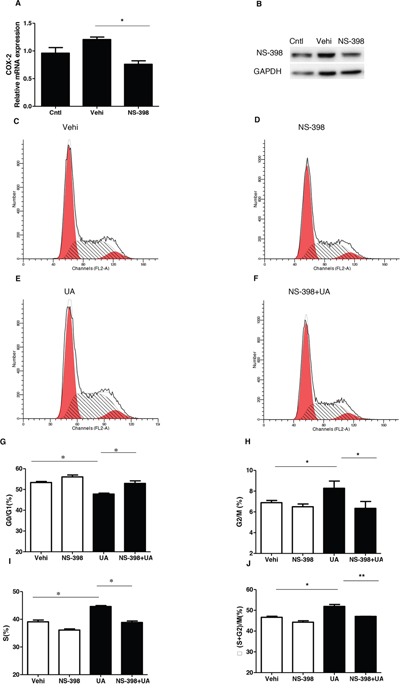
Effect of COX-2 specific inhibitor NS-398 on cell cycle progression after uric acid treatment The cells were treated with NS-398 (10 μM) for 12 h before uric acid (300 μM) administration. **A.** qRT-PCR analysis of COX-2 after COX-2 inhibitor (NS-398) treatment in mesangial cells. **B.** Western blotting analysis of COX-2 expression after COX-2 inhibitor treatment in mesangial cells. **C-F.** Representative images of cell cycle following COX-2 inhibitor treatment with or without uric acid treatment. **G-J.** Percentage of cells at G1/G0 (G), G2/M (H), S (I), and (S + G2)/M (J) following COX-2 inhibitor treatment with or without uric acid treatment. Values are means ± SE; n = 6 in each group. * *P*< 0.05 vs. control, ** *P*< 0.01 vs. control.

**Figure 6:**
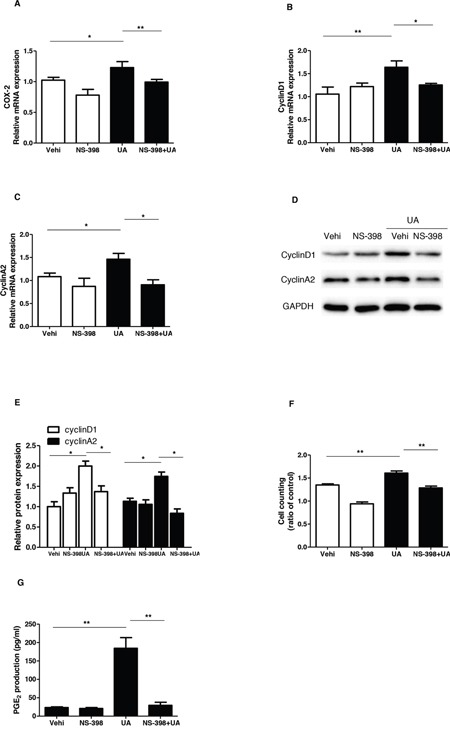
Effect of COX-2 specific inhibitor NS-398 on uric acid-induced mesangial cell proliferation **A-C.** The mRNA analyses of COX-2 (A), cyclin D1 (B), and cyclin A2 (C). Cells were pretreated with NS-398 for 12 h and then were treated with uric acid for another 24 h at a dose of 300 μM. **D.** Representative images of Western blots of cyclin A2 and cyclin D1 after COX-2 inhibition. **E.** Quantification of the Western blots of cyclin A2 and cyclin D1. **F.** Cell counting analysis. **G.** Enzyme immunoassay of PGE2 in the medium. Values are means ± SE; n = 6. * *P*< 0.05 vs. control, ** *P*< 0.01 vs. control.

To evaluate the role of COX-2 in UA-induce MC proliferation, a specific COX-2 inhibitor (NS-398) was applied to the MCs before UA administration. As shown by Figure 5A & 5B, COX-2 inhibitor lowered COX-2 expression at both mRNA and protein levels. Furthermore, COX-2 inhibition was found to decrease the cell number in S phase and increase cell number in G1/G0 phase (Figure 5C-5J). Meanwhile, COX-2 inhibition with NS 398 markedly attenuated UA-induced upregulation of cyclin D1 and cyclin A2 at mRNA and protein levels (Figure 6A-6E). Moreover, cell counting analysis demonstrated an inhibition rate of 32 % of cell proliferation following COX-2 inhibition in UA-treated cells (Figure 6F). These data highly suggested COX-2 played an important role in mediating MC proliferation.

### Inhibiting COX-2 significantly blocked UA-induced PGE_2_ production

To further examine the efficacy of COX-2 inhibition in this experimental setting, we measured PGE_2_ production in the medium. As shown by Figure 6G, UA treatment increased PGE_2_ level by 6.8 folds, which was almost entirely abolished by COX-2 inhibition. The result demonstrated a COX-2-dependent induction of PGE_2_ in response to UA treatment.

### Silencing mPGES-1 blocked UA-induced MC proliferation

**Figure 7:**
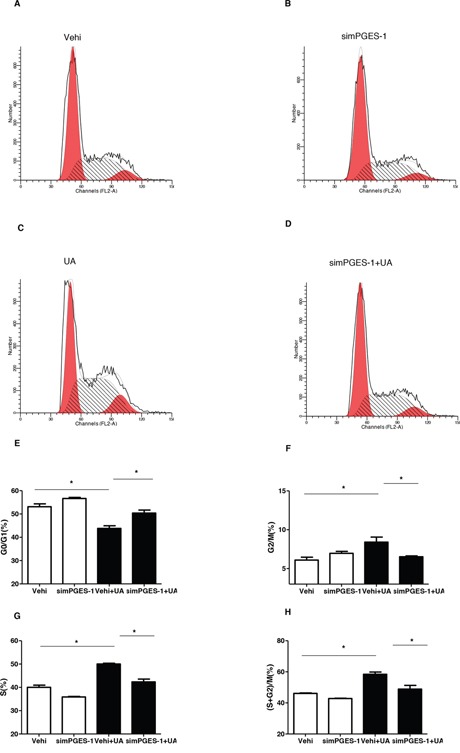
Silencing mPGES-1 ameliorated uric acid-induced cell cycle progression in mesangial cells The cells were transfected with mPGES-1 siRNA for 24 h before uric acid (300 μM) administration. **A-D.** Representative images of cell cycle following mPGES-1 siRNA transfection with or without uric acid treatment. **E-H.** Percentage of cells at G1/G0 (E), G2/M (F), S (G), and (S + G2)/M (H) phases following mPGES-1 siRNA treatment with or without uric acid administration. Values are means ± SE; n = 6 in each group. * *P*< 0.05 vs. control, ** *P*< 0.01 vs. control.

**Figure 8:**
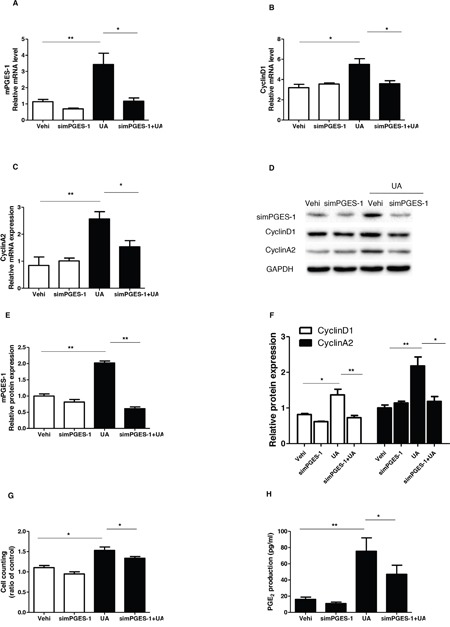
Silencing mPGES-1 blocked uric acid-induced mesangial cell proliferation The cells were transfected with mPGES-1 siRNA for 24 h before uric acid (300 μM) administration. **A-C.** qRT-PCR analyses of mPGES-1 (A), cyclin D1 (B), and cyclin A2 (C). D. Representative images of the Western blots of mPGES-1, cyclin A2, and cyclin D1. **E & F.** Quantification of the Western blots of mPGES-1 (E), cyclin A2, and cyclin D1 (F). **G.** Cell counting Analysis. **H.** Enzyme immunoassay of PGE2 in the medium. Values are means ± SE; n = 6 in each group. * *P*< 0.05 vs. control, ** *P*< 0.01 vs. control.

As a specific PGE_2_-producing enzyme, mPGES-1 chiefly accounts for the inducible PGE_2_ generation. Therefore, mPGES-1 siRNA was applied to the MCs to test the role of mPGES-1 in mediating UA-induced MC proliferation. As shown by the data, the cell cycle progression was significantly blocked as shown by the decreased cell numbers in S and G2/S phases and increased cell numbers in G1/G0 phase following mPGES-1 siRNA treatment (Figure 7A-7H). Furthermore, the upregulation of cyclin D1 and cyclin A2 induced by UA was significantly suppressed by silencing mPGES-1 in UA-treated MCs (Figure 8A-8F). Consistently, cell counting analysis showed that mPGES-1 silencing significantly reduced the cell number after UA treatment (Figure 8G). These data demonstrated that mPGES-1 could be of importance in mediating UA-induced MC proliferation.

### Silencing mPGES-1 blocked UA-induced PGE_2_ production

Finally, we evaluated the effect of mPGES-1 silencing on UA-induced PGE_2_ generation. As shown by the EIA data, mPGES-1 siRNA significantly blocked PGE_2_ production after UA treatment (Figure 8H). This result highly suggested that mPGES-1 was responsible for the induction of PGE_2_ to some extent in response to UA treatment.

### Urinary PGE_2_ assay

**Figure 9:**
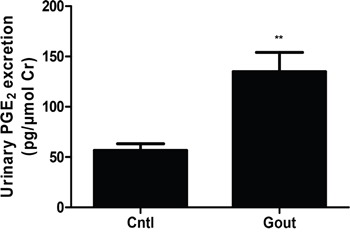
Urinary PGE2 excretion in gout patients and healthy controls The urine samples from 7 gout patients and 11 age-and gender-matched healthy controls were collected and the urinary levels of PGE2 were determined by a ELISA kit. Values are means ± SE; n = 11 in control group and n = 7 in Gout group. ** *P*< 0.01 vs. control.

As shown above, UA could directly induce PGE_2_ secretion in MCs in parallel with cell proliferation, then we measured the levels of urinary PGE_2_ in gout patients and healthy controls. As shown in Figure 9, the urinary PGE_2_ production in gout patients was significantly higher than the controls. This result highly suggested a translational potential of current study to the clinic.

## DISCUSSION

Accumulating evidence demonstrated an association between hyperuricemia and renal diseases [14, 15]. Hyperuricemia could contribute to the glomerular damage like glomerular hypertrophy [16], mesangial cell proliferation [9] and matrix deposition, glomerular sclerosis [17], and tubular interstitial injury [18]. During this pathological process, the activation and proliferation of mesangial cells might serve as an important factor leading to the glomerulosclerosis and the final loss of renal function. Recent report indicated that UA has a direct role in promoting MC proliferation [9]. However, the pathological mechanisms of UA-induced MC proliferation remain elusive. In the present study, we fully evaluated the activation and contribution of a typical pro-inflammatory cascade of COX-2/mPGES-1/PGE_2_ in MCs challenged with UA.

Increasing evidence suggests that proliferation of various types of renal cells including mesangial cells participates in the pathology of renal injury [19, 20]. Cell proliferation is ultimately regulated at the level of cell cycle progression through four stages of G1, S, G2, and M with important checkpoints in G1 and G2 phases. It is well-known that the progression of cell cycle is mainly regulated by cyclins in mammalian cells. For examples, cyclin D1 controls cell cycle progression through the G1 phase and G1-to-Stransition [21], and cyclin A is required for entry into S, completion of S, and entry into M phases [6]. In the present study, our data showed that UA at the doses of 50 to 300 μM directly increased MC number, MC DNA synthesis rate, expressions of cyclinD1 and cyclin A2, as well as the cell number in S phase, indicating a direct effect of UA on promoting MC proliferation as previously demonstrated by our group [9].

It is established that COX-2/mPGES-1/PGE_2_ cascade is of importance for the onset and progression of multiple kidney diseases via pro-inflammatory mechanisms [23]. More interestingly, a recent study showed a regulatory effect of UA on COX-2 expression and PGE_2_ synthesis without functional analysis [23]. Thus, in this study, we firstly examined the regulation of COX-2 and a best-characterized specific PGE_2_ synthase mPGES-1 in mouse MCs following UA treatment. As expected, both COX-2 and mPGES-1 were remarkably elevated by UA in time- and dose-dependent manners. In order to define the role of COX-2 in UA-induced MC proliferation, a specific COX-2 inhibitor (NS 398) was applied to the cells before UA administration. Notably, COX-2 inhibition strikingly blocked UA-induced MC proliferation as evidenced by the reduction of total cell number, the percentage of cells in S and G2/M phases, and the levels of cyclin D1 and A2 in line with a blockade of PGE_2_ production. These data demonstrated a detrimental role of COX-2 in promoting MC proliferation in response to UA challenge.

Next, we examined the contribution of mPGES-1, a downstream enzyme of COX-2 in responsible for the generation of inflammatory mediator PGE_2_ [24], in UA-caused MC proliferation via a siRNA approach. In agreement with the findings from COX-2 inhibitor experiments, mPGES-1 siRNA significantly ameliorated UA-induced MC proliferation in parallel with a significant blockade of PGE_2_ secretion. These results highly suggested that mPGES-1-derived PGE_2_ could play a role in promoting UA-induced MC proliferation as a downstream mechanism of COX-2 in this pathological process.

In addition to the role of pain-triggering, PGE_2_ is also an established mediator of inflammation. A previous study reported that PGE_2_ was involved in the crystal-induced inflammation of gouty arthritis [25]. Here, we examined the urinary levels of PGE_2_ in gout patients and found a significant elevation of urinary PGE_2_ excretion in these subjects as compared with the age-and gender-matched healthy controls. To my knowledge, this is the first clinical evidence demonstrating the enhancement of urinary PGE_2_ in patients with hyperuricemia. Moreover, this result also indicated a potential of clinical translation of current cell-based study.

In summary, using mouse MCs, we identified an activation of COX-2/mPGES-1/PGE_2_ cascade in response to UA challenge. Moreover, employing the pharmacological and genetic approaches, we firstly proved that COX-2/mPGES-1/PGE_2_ cascade activation served as a crucial pathological mechanism in mediating the direct effect of UA on promoting the MC proliferation.

## MATERIALS AND METHODS

### Reagents and antibodies

UA was bought from Sigma (St. Louis, MO). Dulbecco's modified Eagle's medium (DMEM), fetal bovine serum (FBS), penicillin-streptomycin, and trypsin solution (EDTA) were bought from Gibco (Invitrogen, Grand island, NY). COX-2 (catalog no. 160106) and mPGES-1 (catalog no. 160140) antibodies were bought from Cayman Chemicals (Ann Arbor, MI). Cyclin A2 antibody (catalog no. ab7956) was purchased from Abcam (Cambridge, MA). Cyclin D1 antibody (catalog no. 2978) and GAPDH antibody (catalog no. ab9485) were purchased from Cell Signaling Technology (Danvers, MA). The PGE_2_ enzyme immunoassay kit (catalog no. 514010-96) was provided by Cayman Chemicals (Ann Arbor, MI). COX-2 inhibitor NS-398 (catalog no. s1772) was from Beyotime (Shanghai, China).

### Cell culture

A mouse mesangial cell (MC) line HBZY-1 was obtained from the China Center for Type Culture Collection (CCTCC Wuhan, China). Cells were cultured in Dulbecco's modified Eagle's medium (DMEM, Gibco), which supplemented with 10% fetal bovine serum (FBS; Gibco), penicillin (100U/ml) and streptomycin (100 μg/ml), and maintained at 37°C in a humidified 5% CO_2_ atmosphere. After mesangial cells were cultivated to 60%–70% confluence, they were treated with UA for 24 h at different doses (0, 50, 100, 300 μM) with or without a pretreatment of NS-398 (COX-2 inhibitor). siRNAs for mPGES-1 and the silencer negative control were bought from Gene Pharma (Shanghai, China). mPGES-1 siRNA consisted of an RNA duplex containing a sense strand: 5′-GCACACUGCUGGUCAUCAATT-3′ and an antisense strand: 5′-UUGAUGACCAGCAGUGUGCTT-3′. Cells were transfected with siRNA using the Lip2000 Kit (Invitrogen, Carlsbad, CA) 24 h before the experiments were initiated according to the manufacturer's instructions.

### Patients

Urine from seven gout patients who were newly diagnosed as gout in the Affiliated Hospital of Nanjing Medical University (Nanjing, China) and eleven age- and gender-matched healthy controls was collected for the analysis of urinary PGE_2_ levels. The blood uric acid levels of seven gout patients were shown in Table 1. Urinary creatinine was used for the normalization of urinary PGE_2_ excretion. The protocol concerning the use of the patients’ samples in this study was approved by the Human Subjects Committee of Nanjing Medical University. Informed consent was obtained from all participants.

**Table 1:** Clinical information for gout patients

Patients	Sex	Age	Plasma levels of uric acid (μM)
1	Female	79	422.5
2	male	50	451.6
3	male	73	550.8
4	Female	47	401.7
5	male	50	521.5
6	Female	63	469.3
7	male	68	513.6

### Analyses of DNA synthesis rate and cell counting

Mesangial cells were seeded in 96-well plates for [^3^H] thymidine incorporation and in 24-well plates for cell counting with same cell density (3 × 10^4^/ml). To measure the synthesis of DNA, pellets were resuspended into DMEM with 1% FBS and placed in 96-well plates. Cells were incubated with [3H] thymidine(5μCi/ml). Following the indicated treatment, cells were harvested by incubation at 4°C with trichloroacetic acid (5%) followed by solubilization in 0.1 N NaOH. Radioactivity was determined by scintillation counting. To assess cell growth, MCs in 24-well plates were stimulated by the indicated agents, and the cell number was counted with a Z1-Coulter Counter (Luton, UK).

### Cell cycle analysis

MCs were treated with the indicated agents and cultured in DMEM without FBS for 24h. Cells were digested with 0.25% trypsin with EDTA, then washed twice with PBS and fixed in 70% ethanol for at least 2 h at 4°C. After centrifugation, cells were treated with RNase, and stained with propidium iodide by using cell cycle detection kit (KeyGEN, Shanghai, China). The number of cells in different cell cycle phases (G1, S, and G2/M) was detected by flow cytometry (BD FACS Calibur flow cytometer, Bedford, MA), and the data were analyzed with modifit3.0 software.

### Quantitative real-time PCR (qRT-PCR)

Total RNA of MCs was extracted by TRIzol reagent (TaKaRa) according to the manufacturer's protocol. The cDNA was synthesized by a PrimeScript RT reagent Kit (TaKaRa) according to the manufacturer's protocol. All the primers, including cyclin D1 (forward, 5′-CGC CCT CCG TTT CTT ACT TC-3′ and reverse, 5′-GCA GTC AGG GGA ATG GTC T-3′), cyclin A2 (forward, 5′-AAG ATG CCC TGG CTT TTA GTG-3′ and reverse, 5′-TAACATTCACTGGCTTTTCGTCT-3′), Cyclooxygenase-2 (forward, 5′-AGGACTCTGCTCACG AAGGA-3′ and reverse, 5′-TGACATGGATTGGAACA GCA-3′), Microsomal PGE synthase-1 (forward, 5′-AGCACACTGCTGGTCATCAA-3′ and reverse, 5′-CTCCACATCTGGGTCACTCC-3′) and GAPDH (forward, 5′-GTCTTCACTACCATGGAGAAGG-3′ and reverse, 5′-TCATGGATGACCTTGGCCAG-3′) were designed using Primer 5 software (available at http://frodo.wi.mit.edu/) and synthesized by Invitrogen. qRT-PCR was performed with SYBR Premix Ex Taq (TaKaRa) and detected by ABI 7500 Real-Time PCR Detection System (Foster City, CA). The cycling program consisted of a preliminary denaturation (95°C for 10 min), followed by 40 cycles (95°C for 15 s and 60°C for 1 min). Fold changes in expression of each gene mRNA was normalized to GAPDH and analyzed using the delta-delta Ct method.

### Western blotting

At the indicated time points, cell medium was removed and washed by cold-PBS. Then MCs were lysed with lysis buffer containing protease inhibitors and centrifuged. The protein concentration was determined by a Micro BCA protein assay kit (Pierce, Thermo). Cellular proteins (sixty micrograms) were separated by SDS-PAGE and transferred onto polyvinylidene difluoride (PVDF) membranes (Bio-Rad). The membranes were blocked in TBS-T (0.1% Tween 20 in TBS) containing 5% defatted milk for 1h at room temperature, and then incubated with primary antibodies cyclin A2 (1:500), cyclin D1 (1:1000), COX-2 (1:500), and mPGES-1 (1:500) at 4°C for overnight, followed by incubation with HRP-labeled secondary antibodies at room temperature for 1 h. GAPDH was used as an internal standard control. Band intensity was measured using Image J software (NIH, Bethesda, MD, USA).

### Enzyme immunoassay (EIA)

After cell culture medium was centrifuged at 12,000 g for 5 min, the concentration of PGE_2_ in the medium was detected by enzyme immunoassay (Cayman Chemical), according to the manufacturers’ protocol. Urine from patients and healthy controls was collected, centrifuged (10,000r/min, 10 min), then was stored at -80°C. The urinary PGE_2_ levels were measured using the EIA kit mentioned above.

### Statistical analysis

Statistical analysis was conducted using one-way ANOVA followed by a Bonferroni posttest or unpaired student's t test. All the data were presented as means ±SE. *P* <0.05 was considered statistically significant.
